# Special emphasis on bone health management in prostate cancer patients: a prospective longitudinal study

**DOI:** 10.1590/S1677-5538.IBJU.2019.0023

**Published:** 2020-02-20

**Authors:** Ashish Sharma, Rahul Janak Sinha, Gaurav Garg, Samarth Agarwal, Asif Akhtar, Vishwajeet Singh

**Affiliations:** 1 King George's Medical University Lucknow India King George's Medical University, Lucknow, India

**Keywords:** Prostatic Neoplasms, Longitudinal Studies, Population Health Management

## Abstract

**Introduction:**

Use of androgen deprivation therapy (ADT) in carcinoma prostate (CaP) has deleterious effect on bone mineral density (BMD) leading to increase incidence of osteoporosis and skeletal-related events. We evaluated bone health status and impact of bone-directed therapy (BDT) and ADT on BMD in these patients from Jan 2015-Dec 2018.

**Materials and Method:**

Baseline bone health was assessed using Tc-99 MDP Bone scan/ DEXA scan for patients on ADT. Monthly zoledronic acid (ZA) was given to high-risk candidates (T-score ≤2.5 or previous hip/vertebral fracture) or Skel et al. metastatic patients who were receiving ADT. Baseline and follow-up (at 12-months) BMD using DEXA scan at various sites (spine, femur total, femur neck and radius) and subjective improvement in bony pain using Numeric Pain Rating Score after administration of ZA were compared.

**Results:**

A total of 96-patients of locally advanced and metastatic prostate cancer receiving ADT with or without BDT were included in the study cohort. Mean age of presentation was 68.4±15.61 years. Median serum PSA was 32.2±13.1ng/mL. There was significant improvement in mean BMD (T-score) in 64-patients post ZA therapy at 12-months (at femoral total, femoral neck and spine; 0.95, 0.79 and 0.68, respectively) (p <0.05) while there was significant deterioration in mean BMD at 12-months (at spine, femoral neck and femoral total; −0.77, −0.55 and −0.66, respectively) in 32 patients who did not receive ZA and were on ADT (p <0.05). Pain scores significantly decreased in patients after 12-months of ZA use (−2.92±2.16, p <0.01).

**Conclusion:**

Bone-directed therapy (Zoledronic acid) leads to both subjective and objective improvement in bone health of prostate cancer patients on ADT.

## INTRODUCTION

Carcinoma of the prostate (CaP) has the highest incidence of Skel et al. metastases among all urological malignancies. Skel.et al. metastasis in these patients causes some of the most worrisome symptoms, which includes intractable bony pain, pathological fracture, spinal cord compression and paresis (1). Prostate cancer usually occurs in elderly population in which the prevalence of osteoporosis is already common and further use of androgen deprivation therapy (ADT) as a treatment modality in these patients has cumulative deleterious effect on bone mineral density (BMD) leading to increase in osteoporosis and skeletal fracture risk. Development of osteoporosis in these patients appears to increase steadily with duration of ADT with an annual bone loss of 0.6-9.6% and most significant loss occurs within the first year of initiation of ADT. Thus, maintaining the optimal bone health status should be on priority while managing these patients. Unfortunately, this aspect of prostate cancer often remains neglected and there is widespread ignorance amongst medical community about optimum management of bone health in these patients even today (2, 3). Failure to properly screen these patients is detrimental to both quantity and quality of life, given the consistent increase in the life expectancy of CaP patients. The present study was undertaken to study the bone health with special emphasis in prostate cancer patients so that early intervention could be attempted to prevent skeletal related events and thus improve their quality of life.

## OBJECTIVES

To evaluate clinical profile (like demographical characteristics, presenting clinical symptoms, baseline serum prostate specific antigen level, vitamin-D deficiency, Gleason grading, presence and pattern of bony involvement) in patients with prostate cancer who presented to tertiary care institute of a developing nation.

To evaluate the bone health with DEXA scan εt bone scan and to assess the impact of bone-directed therapy (BDT) in improving bone health in these patients.

To reduce skeletal-related events in prostate cancer patients by early intervention thus reducing morbidity and mortality.

## MATERIAL AND METHODS

We recruited consecutive patients of CaP of all age group who presented to the department of Urology at a tertiary care hospital of North India from January 2015 to December 2018. Patients with metabolic or congenital bone disease, prostate secondaries, other active malignancy, central nervous disorders, and moribund status were excluded from the study. Institutional review board clearance and written informed consent from all patients was obtained. This time bound prospective study was registered with Central Trial Registry of India (CTRI) with reference number CTRI/2016/08/007205 (5). Workup and management of CaP patients of study cohort was done as per the European Association of Urology (EAU) guidelines and patient's personal preference (4). Patients were followed every 3 months with serum prostate specific antigen (PSA) levels to look for adequacy of cancer control. Castrate resistant prostate cancer patients were started on the appropriate chemotherapy (docetaxel)/abiraterone/enzulatamide therapy or watchful waiting according to the EAU guidelines and patient preferences.

Baseline bone health was assessed using Tc-99 MDP Bone scan (presence of bony pain, Gleason score >7, serum PSA >10ng/mL and palpable disease cT2/T3) and Dual Energy X-ray Absorptiometry (DEXA) scan for patients on ADT for at least 6-months duration, or presence of clinical risk factors like past history of fracture, excessive alcohol consumption, current smoking and vitamin D deficiency. The BMD was measured by T-score at spine, total femur, femur neck and radius bone in gm/cm^2^. Advanced prostate cancer patients (locally advanced and metastatic) who were on ADT and in whom DEXA scan was indicated and could be obtained, served the final study cohort. Bone-directed therapy (injection of Zoledronic Acid 4mg intravenous infusion monthly+Vitamin D+Calcium Supplementation) was started in these patients of androgen deprivation therapy (ADT) with either positive bone scan or with high fracture risk on DEXA scan (T score ≤2.5) or positive history of previous hip/vertebral fracture. Dose modifications of zoledronic acid (ZA) therapy was done based on renal function status (creatinine clearance>60mL/min: 4mg, 60-40mL/min: 3.5mg, 40-30mL/min: 3mg and if <30mL/min then contraindicated). The baseline and follow-up (at 12-months) BMD at various sites were compared pre and post ZA at spine, femur total, femur neck and radius using DEXA scan. Subjective measurement of pain was done using 11-points Numeric Pain Rating Score (NRS) where 0 indicates no pain, 1-3 indicates mild pain, 4-6 indicates moderate pain and >7-10 means severe pain in patients who received ZA at baseline and after 12-months (6). Bone-directed therapy (injection of Zoledronic Acid 4mg+Vitamin D+Calcium Supplementation) was administered on monthly basis. DEXA scan was repeated after 12 months to look for changes in bone health.

### Statistical analysis

Statistical analysis was performed by an independent statistician using IBM SPSS Statistics ver. 21.0 software (IBM Co., Armonk, NY, USA). T score (BMD) at baseline and after 12 months of therapy was compared at spine, femur neck, total femur and wrist using Wilcoxon Signed Ranks Test. Subjective relief in pain was analyzed by the patient's perception of pain pre and post injection of Zoledronic Acid therapy on an 11-points numeric pain rating scale and compared using the Wilcoxon Signed Ranks test. Bone health of patients who were on ADT but did not receive injection of Zoledronic Acid therapy because of non-compliance, chronic kidney disease or financial constraint were compared with those who were on ADT with Zoledronic Acid (4 mg monthly) administration, using the Mann-Whitney test. P value <0.05 was considered statistically significant.

## RESULTS

**Figure 1:**
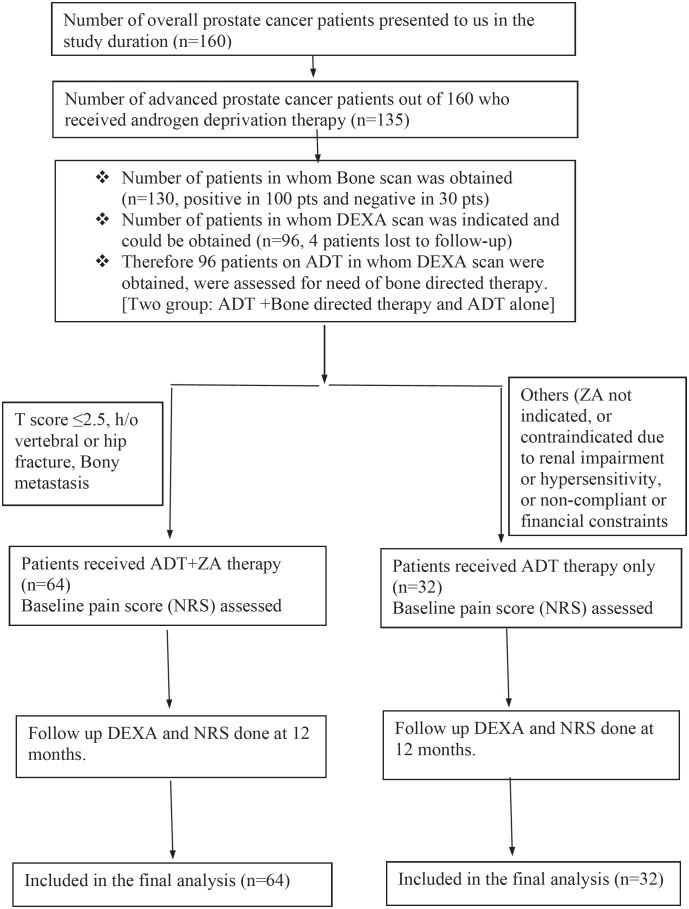
Flow Chart of the study.

We initially enrolled a total of 160 diagnosed prostate cancer patients of all stages. Out of these 160 patients, 135-patients (either locally advanced or metastatic stage) received ADT. Bone scan was obtained in 130 of these patients and it was positive in 100 patients. Ninety six patients with advanced prostate cancer (who were on ADT and in whom DEXA scan was indicated and could be obtained) were included in final study analysis as study subjects. Bone health could not be assessed in other patients due to non-availability of DEXA scan (either not indicated or could not be obtained due to non-compliance or financial reasons or lost to follow-up) and therefore, these patients were excluded from study. This study flow is depicted in Figure-1. Mean age of presentation was 68.4±15.61 years. Median serum PSA was 32.2±13.1ng/mL. Majority of these patients had osteopenia (29.2%) or osteoporosis (64.6%) on DEXA scan. Baseline clinical profile, radiological, pathological and therapeutic characteristics of these patients is depicted in Tables 1 and 2. Zoledronic acid therapy was initiated in 64 of our patients and were followed up with DEXA scan and subjective assessment of bony pain using Numeric Pain Rating Score at 12 months. These were termed as therapy (Bone-directed therapy) group. However, 32 patients were on ADT but did not received Zoledronic Acid therapy due to non-compliance, chronic renal insufficiency status, hypersensivity reactions and financial constraint. These patients served as control or non-therapy group in our study.

**Table 1:** Demographical data and baseline characteristics of prostate cancer patients (n=96).

Baseline Characteristic (N=96)		Number of Patients (N)	Percentage %
**Age Distribution**			
	41-50	9	9.37
	51-60	13	13.54
	61-70	54	56.25
	71-80	17	17.70
	>80	3	3.12
**Geographical Distribution**			
	Rural	37	38.54
	Urban	59	61.45
**ECOG Score**			
	PS 0	72	75.0%
	PS 1	16	16.67%
	PS 2	5	5.2%
	PS 3	2	2.0%
	PS 4	1	1.04%
**Presenting Symptoms**			
	Voiding LUTS	50	52.08
	Bony pain	29	30.20
	Acute urinary obstruction	9	9.37
	Hematuria	2	2.08
	Incidental	6	6.25
**Prostate Size (cc)**			
	20-40	35	36.45
	41-60	27	28.12
	61-80	20	20.83
	81-100	10	10.41
	>100	4	4.16
**Serum PSA**			
	<4.0	2	2.08
	4-20	5	5.20
	20-100	27	28.12
	>100	62	64.58
**Baseline Serum Vit D (ng/mL)**			
	Deficiency (<10)	9	9.37%
	Insufficiency (10-30)	69	71.87%
	Sufficiency (30-100)	18	18.75%
Baseline Characteristics		Average ± SD	Range
BMI (kg/m^2^)		22.5±3.34	15-32
Serum PSA (ng/dL)		32.2±13.1	0.02-2698
Mean ADT Duration (Months)		24.6±13.3	1-147
Mean Follow-Up Duration (Months)		32.8±12.2	8-48
Mean Duration of BDT (Months)		14.6 ±9.6	8-25

**Table 2:** Description of radiological, pathological and therapeutic parameters.

Parameters		Number of Patients (N)	Percentage%
**Biopsy (N=96)**			
	5+4	34	35.41
	4+5	14	14.58
	4+4	16	16.67
	4+3	13	13.54
	3+4	12	12.5
	3+3	7	7.29
**Gleason Grading (N=96)**			
	High Grade (>7)	62	64.58
	low Grade (<=7)	34	35.33
**Baseline DEXA Scan (N=96)**			
	Normal (T Score < −1.0)	6	6.25
	Osteopenia (T Score −1.0 to −2.5)	28	29.17
	Osteoporosis (T Score < −2.5)	62	64.58
**Staging (N=96)**			
	Locally-advanced CaP	04	25.0%
	Metastatic (Skeletal+Visceral)	92 (90+2)	75.0%
**Pattern of Bony involvement (N=90)**#			
	Spine	84	93.33
	Pelvis	72	80.0
	Femur	40	44.44
	Shoulder	27	30.0
	Ribs	45	50.0
	Skull	19	21.11
	Sternum	10	11.11
	Clavicle	3	3.33
Treatment Modality [single/multimodal]*			
	Bilateral Orchidectomy	65	67.7
	LHRH Agonists	8	8.33
	LHRH Antagonists	2	2.08
	Radical Prostatectomy+ADT/RT	2	2.08
	Watchful waiting	3	3.12
	Docetaxel Chemotherapy	2	2.08
	Abiraterone therapy	5	5.20
	Enzalutamide	3	3.12

# Total no. exceeds 100% (n=90) as some patients have >1 bone involvement;

* = Total no. exceeds 100% (n=96) as some patients received more than one treatment modality along study duration

**Figure 2:**
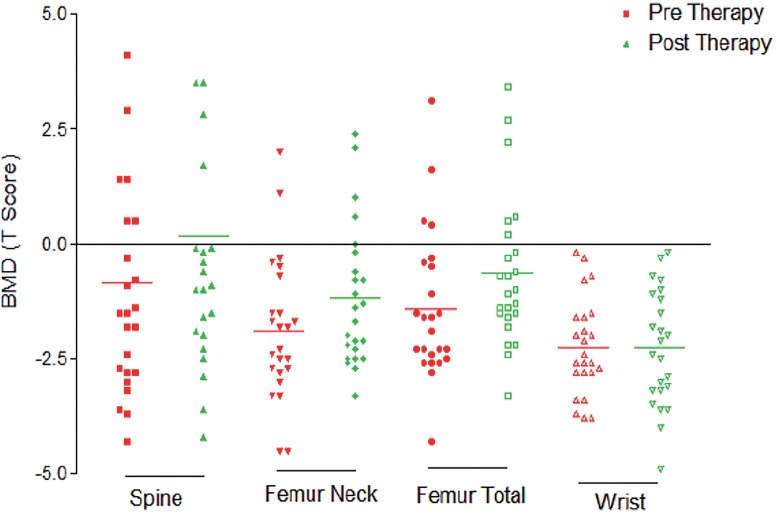
Baseline and post-therapy (Zoledronic acid therapy) BMD (T-Score) showing improvement at all site except radius.

BMD measured as T-Score at various sites was found to improve statistically at all sites (at spine, femur total and femur neck) except radius in patients taking ZA therapy (64 patients) after 12-months (P <0.05) (Table-3, Figure-2). Most significant improvement in mean BMD after instituting Zoledronic acid therapy was noted in femoral total followed by femoral neck and spine (0.95, 0.79 and 0.68, respectively).

**Table 3:** Comparison of baseline and follow up BMD at various bone sites and Pain Scores with different therapeutic modalities.

BMD (T Score)	Baseline (Mean±SD)	Follow-Up (Mean±SD)	Difference (Mean±SD)	Z Score	P Value
**Impact of ADT on BMD in patients receiving Bone-directed therapy (Zoledronic acid) at various bone sites (N=64)**					
Spine	-0.91±1.62	0.59±2.10	0.68±1.12	-3.45	0.001*
Femur Neck	-1.90±1.43	-1.11±1.64	0.79±0.97	-2.89	0.002*
Femur total	-1.30±1.53	-0.55±1.02	0.95±0.91	-3.91	0.000*
Radius	-1.18±1.01	-1.24±1.32	0.06±0.77	-1.12	0.361
**Impact of ADT on BMD in patients without Bone-directed therapy (Zoledronic acid) at various bone sites (N=32)**					
Spine	-1.03±1.17	-1.80 ±1.50	-0.77±0.99	-2.62	0.012*
Femur Neck	-2.04±1.23	-2.70± 1.63	-0.65±0.75	-2.50	0.015*
Femur Total	-1.63±1.21	-2.18±1.57	-0.55±0.65	-2.63	0.005*
Radius	-1.06±1.43	-1.21±1.55	-0.15±0.17	-1.14	0.154
**Impact of Zoledronic acid therapy on Bony Pain measured by Numeric Pain Rating Score (NRS) on 0-10 Scale (N=96)**					
ZA Therapy Group (N=64)	5.11±1.80	2.19±1.34	-2.92±2.11	-3.53	0.0001*
Non-therapy Group (N=32)	2.81±1.30	4.41±2.45	+1.60±1.72	-2.26	0.020*

* P value<0.05

**Figure 3:**
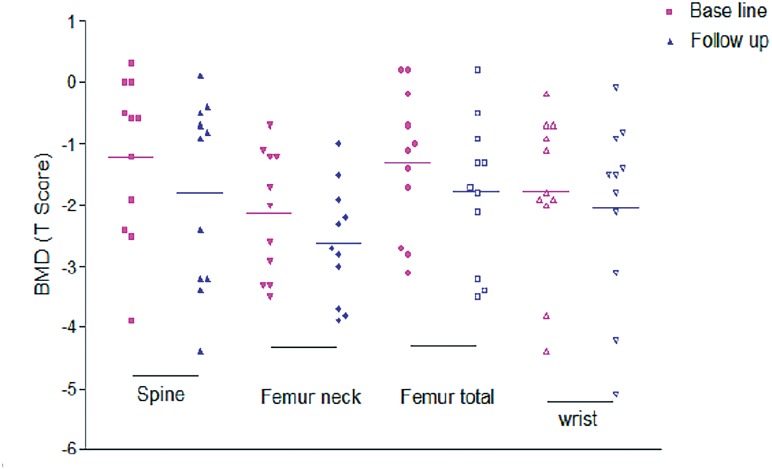
Baseline and Follow-up BMD (T-Score) in patients on ADT without Zoledronic acid therapy showing worsening at all site except radius.

**Figure 4:**
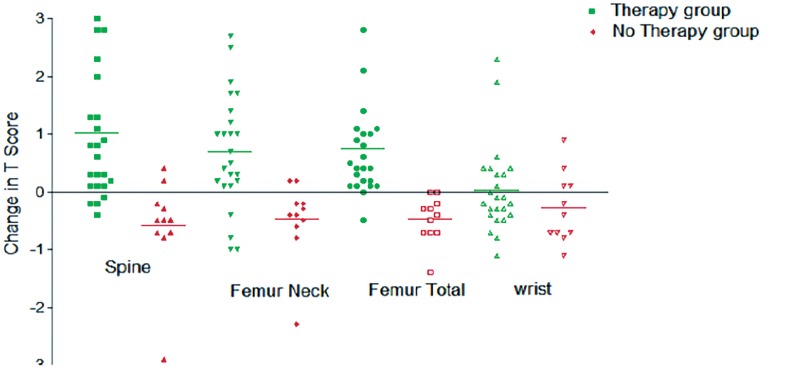
Comparison of T-Scores in patients who received therapy and did not received therapy showing favourable results for therapy group.

However, BMD changes after 12 months in patients on ADT and not receiving ZA therapy (32 patients) showed significant decrease in T score at all sites except radius (P <0.05) (Figure-3). Most significant deterioration in mean BMD in this group of patients was noted in spine followed by femoral neck and femoral total (−0.77, −0.55 and −0.66, respectively) (Table-3, Figure-4).

**Figure 5:**
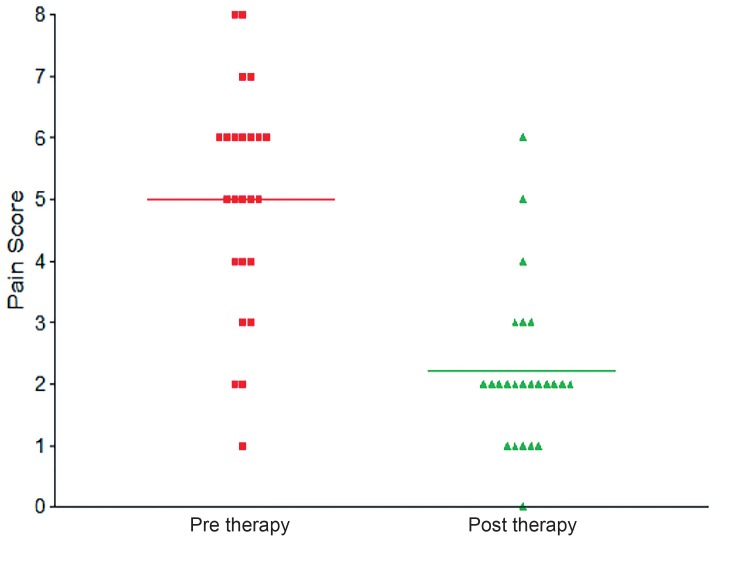
Comparison of pain scores pre and post Zoledronic acid therapy in patients on ADT showing a significant improvement in pain scores.

Pain scores significantly decreased in patients after 12-months of ZA use (−2.92±2.16, p <0.01) (Table-3, Figure-5). Pain Scores in patient on ADT not receiving the ZA therapy showed a significant deterioration at follow-up from a baseline of 2.81±1.32 to 4.41±2.54 at mean with a change of +1.60±1.88 (p=0.002).

Skeletal related events (spinal cord compression, need for radiotherapy or surgery for bony metastasis) were seen in 30 out of 160 (18.7%) enrolled patients during the four years of the study duration. Fracture rates were significantly lower in bone-directed therapy group (5.5%) as compared to non-therapy group (12.8%). (Relative risk: 0.29, 95% confidence interval: 0.28-0.41). Multi variate analysis (using Cox proportional hazards regression model) results showed that advanced age >70 years (HR-1.89, p=004), Gleason score ≥7 (HR-1.52, p=0.013), presence of bony pain (HR-2.80, p <001), presence of extra-spinal skeletal metastasis (HR-1.96, p=0.036) and absence of bone-directed therapy (HR-2.6, p=0.002) were associated with the risk of skeletal related events in prostate cancer patients. Twenty-four (25.0%) patients of study cohort died within the follow-up period of 4 years.

Zoledronic acid treatment was well tolerated by most of our subjects except 5 patients who developed mild fever and 4 patients who developed reversible multiple joint pain with fever which lasted for 1-2 days. These patients were treated with oral Paracetamol and recovered well. They did not discontinue the therapy. None of the patients developed osteonecrosis of the jaw in the current study. Zoledronic Acid not only prevents bone mineral density loss but also improves BMD, thus decreasing the fracture risk and diminishing the patient's perception of pain.

## DISCUSSION

The present study from India is a sobering reminder of prostate cancer presentation in absence of PSA screening: Of all 160 patients, 63% had PSA >100ng/mL, approximately 92% patients with PSA >20ng/mL, 50% patients had Gleason 9, 93% patients had Gleason 7 or higher with much higher frequency of metastatic disease and use of bilateral orchiectomy as ADT modality. This study demonstrates that ZA therapy improved BMD and resulted in less pain and fewer skeletal events. It also highlights that ADT associated bone loss is underreported and relatively neglected even in the present era.

The largest percentage (48.12%) of our total 160 patient cohort were between 60-70 years age group followed by 21.88% patients between 70-80 years group hence making it around 75% population greater than 60 years with average age being 68.4 years. This is in concordance with literature which stats that more than three quarter of cancer prostate occur after the age of 65 years (7), somewhat lower than median age of 72 years in another series (8). We had no patient under 40 years of age and only 10.6% were under 50 years suggesting that it is quite uncommon below the age of 50 years. The average age of death from carcinoma prostate is 77 years and has remained stable over the last three decades (9). Similarly in our series, we saw few patients (4.37%) in the age group above 80 years. It can also be explained by the fact that average life expectancy in India as per World Health Organisation, 2015, is around 66.9 years for men, thus, not many patients live long enough to present with the disease after 80 years of age.

The positive DRE finding in almost all our cases represent the late stage of presentation of the disease in this part of the world. In western countries, after the introduction of PSA testing, 81% of newly diagnosed men have localized disease, whereas the incidence of metastatic disease has decreased by 75% (10). Non-palpable cancers (AJCC clinical stage T1c) now account for 60-75% of newly diagnosed cancers (11). This is in contrast to the findings seen in our patients. By the time the patients present to us, they already have hard prostate and the diagnosis in almost all cases can be easily reached by simple digital rectal examination. As previously mentioned, around 20% patients come to us with bony pain as their first presenting symptoms, signifying lack of screening programme for cancer prostate in our country.

Incidence of bony metastases in prostate cancer patients is quite high in India. Majority (68.7%) of our study subjects (110 out of 160) were metastatic (on either bone scan/MRI/CECT scan) at presentation, while in western population about 80% of patients have localized disease at presentation (10). It shows that most of our patients have higher stage and grade at presentation and hence indicates poor prognosis, high morbidity and mortality at the time of diagnosis itself (Table-2). The most common site of metastases observed in our study was the spine in 93% cases followed by pelvis in 80% cases.

ADT is commonly used in our setting because most of the patients present in advance stage and are not the candidates for radical prostatectomy. Most common treatment modality used in our setting was bilateral orchidectomy followed by GnRH agonists. Most of the study subjects were from poor socioeconomic background and could not afford costly treatment of GnRH agonist and antagonist and hence chose bilateral orchidectomy. Androgen deprivation associated bone loss is an increasingly prevalent and important consideration in patients with prostate cancer. In our patient population we found that baseline DEXA scores of 96 patients suggested osteoporosis (T--Score <-2.5) in 64.5% cases and osteopenia (T--Score between −1.5 to −2.5) in 29% cases leaving a small percentage of patients (6.2%) with normal bone health status. A study by Agarwal et al. in Indian patients showed significant loss of bone mineral density after orchidectomy (13% at 6 months and 18% at 1 year), which results in an increased incidence of osteoporosis from 24-48% at 6 months after performing orchidectomy (12). Bone health in cancer prostate is often a neglected aspect even in present era especially in developing nations.

The current study shows that there was significant improvement in mean BMD (T-score) in 64-patients post ZA therapy at 12-months (at femoral total, femoral neck and spine, 0.95, 0.79 and 0.68, respectively) (p <0.05) while there was significant deterioration in mean BMD at 12-months (at spine, femoral neck and femoral total, −0.77, −0.55 and −0.66, respectively) in 32 patients who did not receive ZA and were on ADT (p <0.05). Kapoor et al., 2011, (13) concluded in a study of 41 patients with non-metastatic cancer prostate patients on ADT, that 3 monthly administration of Zoledronic acid for 1 year improved vertebral and left femoral neck BMD in men on GnRH-agonist treatment. They noticed that alteration in vertebral and left femoral neck BMD was significantly higher in Zoledronic acid therapy group than in the placebo group. Saad et al. (14) studied the effect of Zoledronic acid therapy on skeletal complications in 643 carcinoma prostate patients with bony metastases. Administration of ZA (4mg or 8mg, 3 weekly) reduced the fracture events and increased the median time to first SRE in their study. We also found that administration of monthly Zoledronic acid (4mg) decreased the SRE in our cohort as compared to non-therapy group (P <0.002).

The pain score was measured at baseline and follow-up showed a decreased from mean of 5.1 at baseline to around 2.2 at follow-up i.e. a 2.9 point improvement in subjective perception, from moderate to mild pain in patients who received injection of Zoledronic Acid. When the group which did not receive the therapy was compared at baseline and follow-up, there was significant deterioration in their pain levels with pain scores showing an increase from a mean of 2.81 to 4.41 at the time of follow-up. When the change in pain score between the treatment groups was compared with no treatment, it was found to be significantly better in the therapy group. A Cochrane review of 3682 patients with substantial proportion of pain relief pooled data (eight studies) demonstrated the benefits in the Zoledronate treatment group (15). Study by Saad et al. as mentioned previously showed that patients who received Zoledronic acid had lower mean pain scores (Brief Pain Inventory composite score) as compared to placebo group at every time point and these differences were found to be statistically significant at the 3 and 9 months time points.

There are few limitations in the present study. The study measured bone health and pain scores till 12-months duration and depict data from a single centre only. Secondly, it was not a randomized study and patients with higher risk were the ones who received ZA. Thirdly, follow-up and compliance to treatment is poor amongst few patients in the study due to complicated disease dynamics in a developing country with poor socio-economic status, lack of nearby healthcare facilities and lack of medical insurances. Health care provider and treating physician should be aware of these obstacles in developing and underdeveloped population and all these factors need to be addressed by concerned authority for improving both quantity and quality of these patients.

To the best of our knowledge, this is the first study from India which focuses on bone health in cancer prostate patients and has studied the effect of Zoledronic Acid in Indian population. Bone health becomes all the more important for this population subgroup as we found that most of the patients presenting to us have a high burden disease with very high PSA levels, high grade disease and positive bony metastases and needs further emphasis for improving their overall quality of life.

## CONCLUSIONS

Bone-directed therapy (Zoledronic acid) improves the bone health of the patients both objectively and subjectively as assessed by DEXA Scan and pain scores, respectively. Since major cause of morbidity in prostate cancer is bony metastases, bone health must be taken in to account in routine clinical practice as per the standard guidelines.
